# Evolutionary origins and development of saw-teeth on the sawfish and sawshark rostrum (Elasmobranchii; Chondrichthyes)

**DOI:** 10.1098/rsos.150189

**Published:** 2015-09-02

**Authors:** Monique Welten, Moya Meredith Smith, Charlie Underwood, Zerina Johanson

**Affiliations:** 1 Department of Earth Sciences, Natural History Museum, London, UK; 2 Dental Institute, Tissue Engineering and Biophotonics, King's College London, University of London, London, UK; 3 Department of Earth and Planetary Sciences, Birkbeck, University of London, London, UK

**Keywords:** chondrichthyes, dermal denticles, rostrum denticles, evolution of teeth, regeneration

## Abstract

A well-known characteristic of chondrichthyans (e.g. sharks, rays) is their covering of external skin denticles (placoid scales), but less well understood is the wide morphological diversity that these skin denticles can show. Some of the more unusual of these are the tooth-like structures associated with the elongate cartilaginous rostrum ‘saw’ in three chondrichthyan groups: Pristiophoridae (sawsharks; Selachii), Pristidae (sawfish; Batoidea) and the fossil Sclerorhynchoidea (Batoidea). Comparative topographic and developmental studies of the ‘saw-teeth’ were undertaken in adults and embryos of these groups, by means of three-dimensional-rendered volumes from X-ray computed tomography. This provided data on development and relative arrangement in embryos, with regenerative replacement in adults. Saw-teeth are morphologically similar on the rostra of the Pristiophoridae and the Sclerorhynchoidea, with the same replacement modes, despite the lack of a close phylogenetic relationship. In both, tooth-like structures develop under the skin of the embryos, aligned with the rostrum surface, before rotating into lateral position and then attaching through a pedicel to the rostrum cartilage. As well, saw-teeth are replaced and added to as space becomes available. By contrast, saw-teeth in Pristidae insert into sockets in the rostrum cartilage, growing continuously and are not replaced. Despite superficial similarity to oral tooth developmental organization, saw-tooth spatial initiation arrangement is associated with rostrum growth. Replacement is space-dependent and more comparable to that of dermal skin denticles. We suggest these saw-teeth represent modified dermal denticles and lack the ‘many-for-one’ replacement characteristic of elasmobranch oral dentitions.

## 1. Introduction

Sharks and rays have been studied extensively to address the origin and evolution of teeth (e.g. 1). These groups, along with the chimaeroids, comprise the living representatives of the Chondrichthyes, a group that forms the sister clade to all other extant jawed vertebrates. Teeth and tooth-like structures are readily observed in sharks and rays; in addition to true teeth present along the jaws, dermal denticles are present in most species, being present both on the skin and in the oro-pharyngeal cavity. Many denticles may be highly modified, as in the case of dermal thorns of skates, tail spines of stingrays and gill rakers of the basking shark. In addition, several living, and at least one extinct, groups of chondrichthyans have tooth-like structures along the lateral margins of an expanded anterior cartilaginous rostrum.

In vertebrates, dermal denticles and oral teeth form initially as odontodes, dentinous structures derived from the interaction between an epithelium and underlying ectomesenchyme. Although the homology of the odontode is not contentious, the evolutionary relationship between external dermal denticles and the oro-pharyngeal dentition remains under discussion (recently reviewed by Donoghue & Rücklin 2, Smith & Johanson 3 and Witten *et al.* 4). The classic ‘outside–in’ hypothesis proposed that oral teeth were derived from the external skin denticles through a heterotopic evolutionary shift into the oro-pharyngeal cavity, from which the functional dentition on the jaw was derived. This implies that denticles and oral teeth share a common developmental ‘toolkit’, not only as morphogenetic units (odontodes), but also including the spatio-temporal patterning and ordered replacement that characterizes the dentition. Thus, denticles on the skin surface would retain the potential to be patterned (e.g. 5; 6; 7; 8; 9) and to be replaced on a regular basis and in advance of function, as in an ordered set of teeth.

A potential test of this is represented by the notably tooth-like denticles on the extended rostrum-saw of sawfish (Pristidae; Batoidea), fossil sclerorhynchids (Sclerorhynchoidea; Batoidea) and sawsharks (Pristiophoridae; Selachii). These ‘saw-teeth’ (previously described as ‘saw-tooth scales’ 10) are arranged in lateral rows and are believed to be used for prey capture and feeding 11; 12; 13. They extend caudally for the length of the rostrum from its tip, and in sawsharks and sclerorhynchids, are continuous with other tooth-like structures in the skin lateral to the chondrocranium.

Our goal is to describe these saw-teeth and evaluate whether they replicate the ordering of oral dentitions, including spatio-temporal arrangement during development, growth and replacement. If so, this would provide a potential evolutionary mechanism for the ‘outside–inside’ hypothesis, whereby the oral dentition was derived from external denticles, through co-option of these denticles, including shared development by means of heterotopic transfer (e.g. of relevant genes or gene networks *sensu* 14).

## 2. Material and methods

Specimens were scanned using the Metris X-Tek HMX ST 225 X-ray computed tomography (XCT) scanner at the Imaging and Analysis Centre, Natural History Museum, and GE Locus SP XCT Tech scanner at the Dental Institute, King's College, London. Three-dimensional renderings, segmentation and analyses were performed using Avizo Standard software (v. 8.0.1) (http://www.vsg3d.com/avizo/standard), VG Studio Max v. 2.0 (http://www.volumegraphics.com/en/products/vgstudio-max.html) and Drishti (http://sf.anu.edu.au/Vizlab/drishti). Macrophotographs were taken on a Leica MZ95 and processed using Leica Application Suite. Measurements were compiled using the three-dimensional length measurement feature in Avizo (electronic supplementary material).

Taxa examined included dry skeletal (A, Royal College of Surgeons, London (Hunterian Museum)), wet preserved (BMNH, Natural History Museum, London, Life Sciences) and fossil specimens (NHMUK P., Natural History Museum, London, Earth Sciences). Wet preserved specimens were mostly fixed in formalin, sometimes first in alcohol, before storage in alcohol. Specimens examined include: Elasmobranchii; Pristiophoridae (sawsharks): *Pliotrema warreni* (BMNH1986.5.9.2) embryo (total length, (TL: from snout to tip of longer caudal fin lobe) 18.8 cm); *Pristiophorus nudipinnis* (BMNH1905.3.28.13) embryo (TL 30.7 cm); *Pr. nudipinnis* (Charlie Underwood (CU) unregistered specimen) adult specimen (rostrum and head approx. 30 cm); *Pristiophorus cirratus* embryos (BMNH1914.8.20.1, TL 29.4 cm; BMNH unregistered specimen, Haslar collection TL 30.2 cm); *Pr. cirratus* (A.439.1) adult (rostrum length approx. 32 cm); *Pristiophorus lanae* juvenile (ZJ unregistered specimen, rostrum and head 18.6 cm).

Elasmobranchii; Batoidea; Pristidae (sawfish): *Anoxypristis* *cuspidata* (A.442.6) neonate specimen (rostrum length 12 cm); *Pristis* sp. (CU unregistered specimen, rostrum length approx. 32 cm).

Elasmobranchii; Batoidea; Sclerorhynchoidea:*Sclerorynchus atavus* adult (NHMUK PV P.4776 (incomplete rostrum, length 16.5 cm); NHMUK PV OR83663 (incomplete rostrum, length approx. 15 cm)).

## 3. Comparative terminology

Dermal denticles (placoid scales) are a micromeric form of external skeleton found in all chondrichthyans and an exclusively fossil group of jawless fish (Thelodonti). These are non-growing, not attached to bone, and are either replaced, or new ones added with increase in body size as they become separately spaced within the skin 1; 10. Their development and structure (as an odontode) is homologous with a single tooth, including the bony base that links into the dense, fibrous tissue of the dermis, whereas teeth link to the fibrous perichondrium around the jaw cartilage. We will refer to all odontodes on the extended rostrum as rostral denticles (or saw-teeth, rather than ‘saw-tooth scales’) and describe them by three simple topographic terms: lateral rostral, ventral rostral or lateral cephalic. Measurements of all developing rostral denticles, both initial from the growing rostrum tip (initial rostral denticles) and replacement along the rostrum (replacement denticles), relative to those that are functional, will provide quantitative data to identify comparable denticles in the embryo and adult (electronic supplementary material).

## 4. Results

As noted above, specimens examined were fixed in formalin prior to storage, which can affect the density of mineralized tissues. However, our prediction is that older individuals would show more mineralization, as would older elements in an individual (proximal versus rostral tip of rostrum, proximal saw-teeth versus more distal, saw-teeth developing under the skin versus laterally erect and functional saw-teeth), and this is what we observe; degree of mineralization varies in a predicted manner. Thus, we assume that levels of mineralization reflect increasing maturity of the matrix and therefore, stages of development can reasonably be extrapolated between embryos and among adult rostra.

## 5. Pristiophoridae (sawsharks; Selachii)

### 5.1 Developmental growth data for denticle initiation

**Figure 1.**
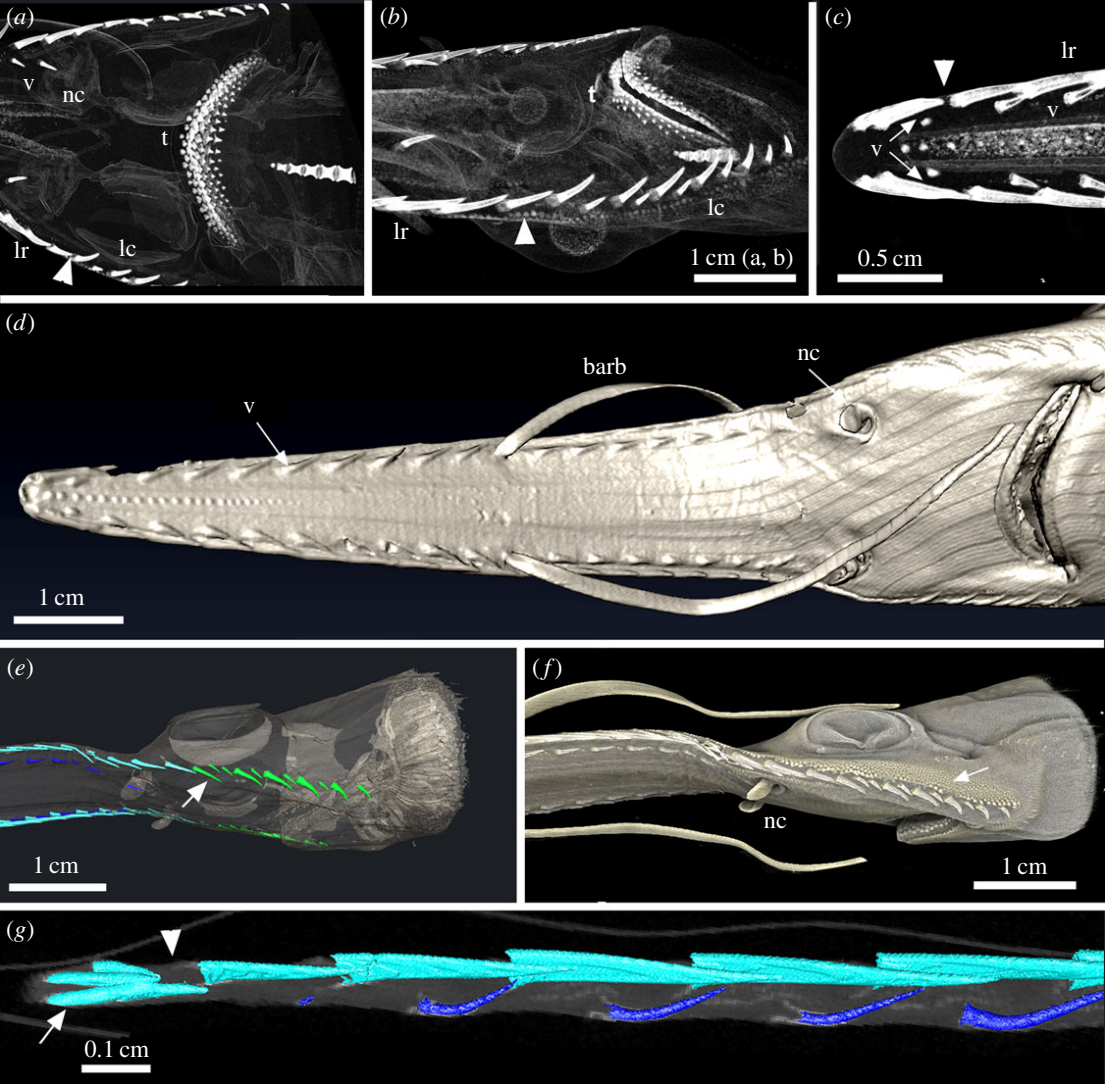
(*a*–*c*) *Pristiophorus cirratus* (Pristiophoridae; Selachii) BMNH1914.8.20.1 (embryo). Volume-rendered Drishti, density selection for mineralized tissues. (*a*,*b*) Topographic pattern of oral dentition compared to the proximo-distal rostrum saw denticles (lateral rostral and lateral cephalic; arrowhead marks transition between them, figure 10*e*); (*c*) rostrum tip, with sites of mineralization of newest ventral denticles, showing size-specific development of crown in each series (arrowhead indicates gap between denticles at rostrum tip and more proximal denticles, as in (*g*). (*d*–*g*) *Pristiophorus nudipinnis* (Pristiophoridae; Selachii) BMNH1905.3.28.13 (embryo). (*d*) Ventral view of head and rostrum, developing denticle series covered by skin; (*e*–*g*) rostrum in ventrolateral views (Avizo, Drishti segmented volume renders). (*e*) Denticle series of lateral rostral (light blue), ventral (dark blue) and lateral cephalic (green; arrow indicates transition between lateral rostral and lateral cephalic series); (*f*) skin denticles developing alongside lateral cephalic denticles (arrow). (*g*) Right lateral rostral and ventral series showing crowding of developing terminal pair of lateral rostral denticles at the tip (white arrow; arrowhead indicates gap between these and larger lateral rostral denticles). The figure is arranged with proximal to the right, dorsal to the top. v, denticles of the ventral series; nc, nasal capsule; t, oral teeth; lr, lateral rostral series of denticles; barb, barbels.

**Figure 2.**
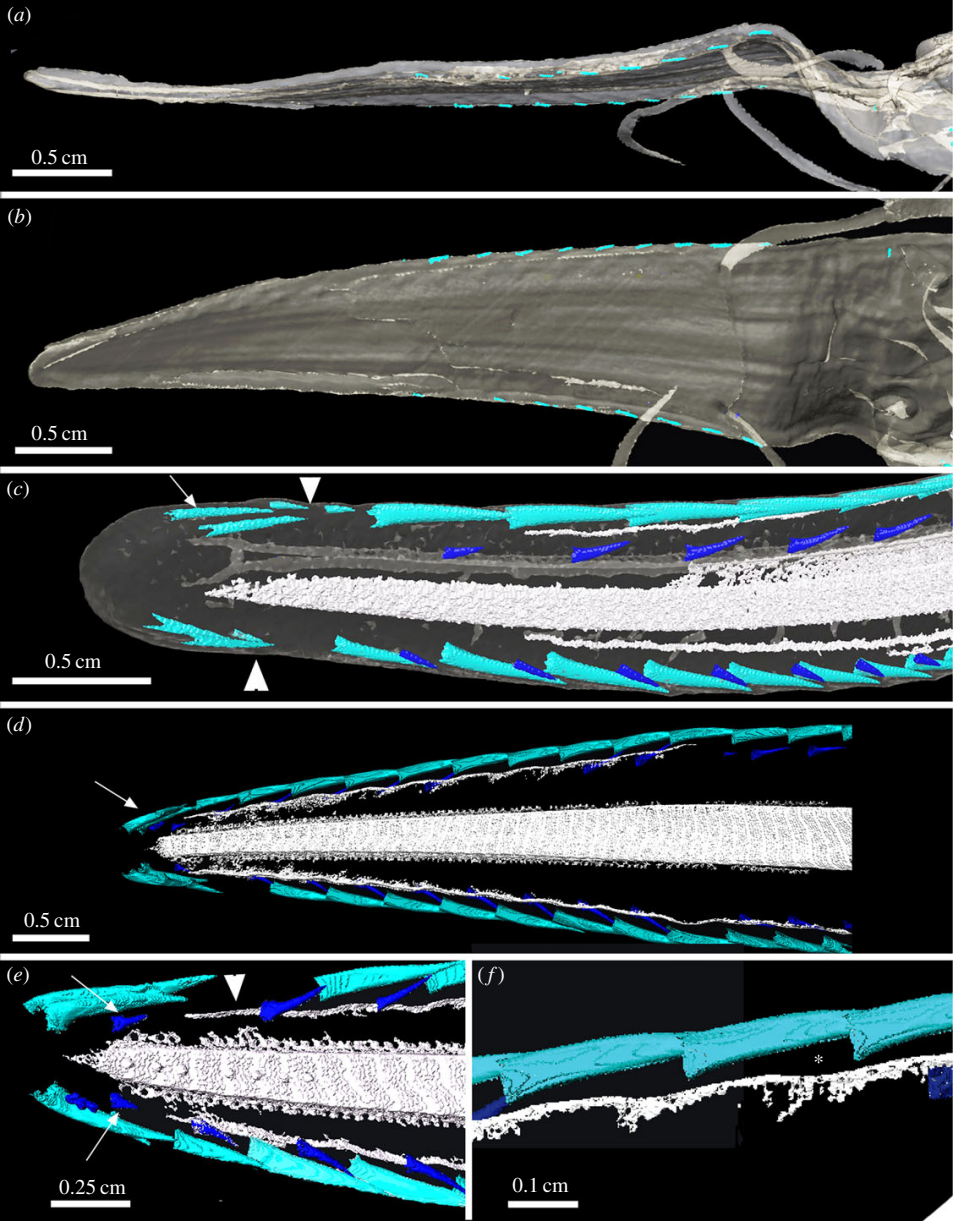
(*a*,*b*) *Pliotrema warreni* embryo (Pristiophoridae; Selachii) BMNH1986.5.9.2 (embryo, volume rendered and segmented with Avizo), in lateral (*a*) and ventral views (*b*), proximo-distal progress in mineralization of lateral rostral denticles (light blue); (*c*) *Pr. cirratus* (Pristiophoridae; Selachii) BMNH1914.8.20.1 (embryo), mineralization of denticles at tip prior to mineralization of the lateral rostrum cartilages (arrow, terminal denticle pair; arrowhead, gap between these and developing lateral rostral denticles); (*d*–*f*) *Pr. nudipinnis* BMNH1905.3.28.13 (embryo). (*d*) Lateral supporting cartilages are mineralizing alongside denticle bases (arrow indicates developing terminal pairs of denticles); (*e*,*f*) shallow depressions in supporting layer of prismatic cartilage along the rostrum below lateral rostral denticles ((*e*) arrowhead, small arrows indicate mineralizing developing ventral denticles at rostrum tip; (*f*) asterisk; comparable depressions in adult, figures 3*g* and 10*a*,*b*). The figure is arranged with proximal to the right, dorsal to the top.

In the embryo (*Pl. warreni*, *Pr. nudipinnis*, *Pr. cirratus*), the rostrum shows expansive growth, with many of the first set of denticles covered by skin (figures 1*d* and 2*a*,*b*). The youngest embryo available, of *Pl. warreni*, demonstrates that the first denticles to develop belong to the lateral rostral series. As noted, they are detected by degrees of mineralization depending on their developmental age (as judged by density differences in rendered XCT-scans); older denticles begin mineralizing caudally, near the barbels, and progressively mineralize towards the distal rostrum (figure 2*a*,*b*). In these denticles, mineralization of the dental tissues proceeds from the tip of the crown, towards the base.

Under the skin of older embryos (e.g. *Pr. nudipinnis*), three sets of denticles are seen to be developing (figures 1*d*–*f* and 2*c*–*f*): the lateral series present in the earlier embryos (figures 1*a*,*b*, lr; 1*e*,*g* and 2, light blue), a second series of smaller denticles on the ventral surface of the rostrum (figures 1*a*,*c*,*d*, v; 1*e*,*g* and 2, dark blue), extending rostrally from the nasal capsules (nc, figure 1*a*,*d*,*f*) and a third series extending laterally along the head to the jaw joint (lateral cephalic, figure 1*a*,*b*, lc; *e*, green). Within the lateral rostral and ventral series, denticles are of equivalent size (ventral are smaller and recurved), oriented rostrocaudally and laterocaudally (figures 1 and 2). In both the lateral rostral and ventral series, the denticles appear equally spaced along the rostrum, especially in the ventral series, with overlap of denticles in the lateral rostral series (figures 1*e*,*g* and 2*c*–*f*). Notably, denticles of the lateral rostral series are not evenly spaced at the tip of the rostrum, where the pairs of each side are close together, one laying on top of the other (figures 1*c*,*g* and 2*c*,*d*, white arrows, 2*e*). There is also a distinct gap between these overlapping denticles and more caudal denticles, in this gap newly developing denticles have a smaller mineralized crown, as the soft tissues of the tooth germ mineralize from the crown tips (e.g. figure 2*c*, white arrowheads). Small ventral denticles are formed close to the cluster of terminal denticles, first as tiny, mineralized crown tips (developing in the soft tissue denticle germs; figures 1*c* and 2*e*, arrows).

Mineralization of the rostral cartilage progresses rostrally (figure 2*c*–*e*), forming shallow depressions for denticle support, coincident with the lateral rostral denticle series (figure 2*d*–*f*, asterisk). These depressions develop prior to the lateral eruption and function of these denticles. It is however notable that the lateral and ventral denticles develop at the rostrum tip prior to cartilage mineralization (figure 2*c*–*e*). In the lateral cephalic series, along the side of the head, denticles are more curved and of two distinct sizes, with the smaller located between the larger; all are oriented medially (figure 1*a*–*f*) and are not supported by the cartilaginous rostrum. The lateral cephalic series is distinct from the lateral rostral, with the transition between the two clearly indicated (figure 1*a*,*b*,*e*, white arrows or arrowheads).

### 5.2 Comparative measurements between fetal and adult

**Figure 3.**
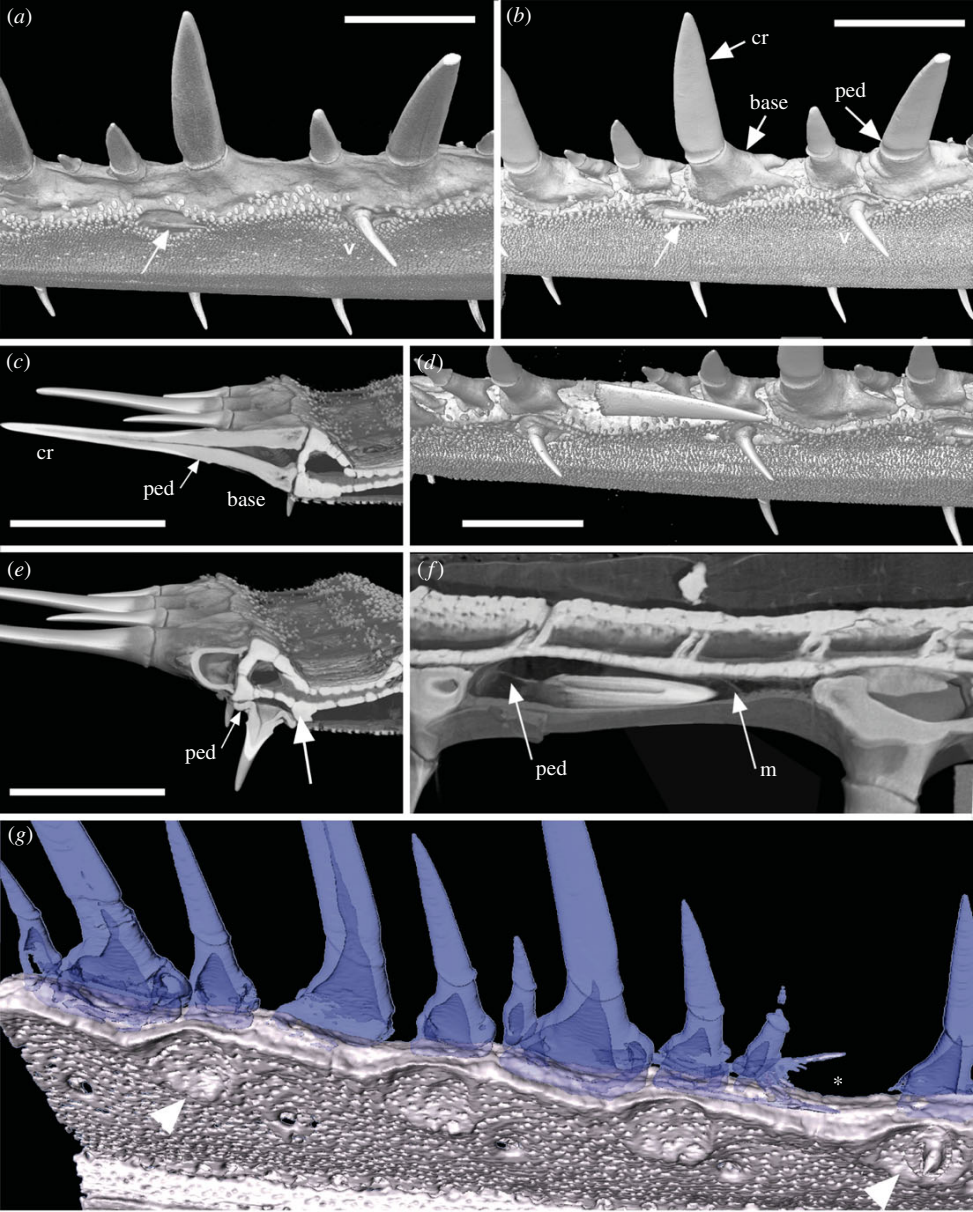
*Pristiophorus nudipinnis*, *Pr. lanae* (Pristiophoridae; Selachii, CU unregistered specimen). (*a*,*b*,*d*) Drishti volume rendered, rostrum showing skin around rostral denticles, oblique lateral views. (*b*) Virtual dissection to remove skin, to show underlying developing ventral denticles (arrows), still beneath the skin (*a*) and exposed within shallow depression (*b*). (*d*) Ventrolateral view of denticle ‘triplet’ in the lateral rostral series and denticles of the ventral series. Crown of replacement large denticle lies flat beneath the skin (virtually removed), above the empty shallow pit of the lost denticle, within a gap between other denticles. (*c*,*e*) Virtual coronal sections. (*c*) Through the base of a large, lateral denticle, with a pedestal, and base above solid prismatic cartilage, as support for attachment. (*e*) Through the base of a ventral denticle showing the pedestal and the pit (white arrow) in the ventral surface of the cartilage. (*f*) Virtual horizontal section showing the developing crown with unmineralized pedestal and a membrane covering the crown tip (m). Mineralized canals can be seen passing from the central cartilage of the rostrum to the lateral cartilage bearing denticles. (*g*) False colour image (Avizo), showing circular depressions as pits (arrowheads, see figure 10*a*,*b*) of the ventral series, also shallow embayments for the largest lateral rostral denticles (asterisk). Scales (*a*,*c*,*d*) 0.5 cm; (*c*,*d*) 0.25 cm. The figure is arranged with proximal to the right, dorsal to the top. cr, crown; base, basal tissue of denticle for attachment; ped, pedestal; m, medium-sized denticles.

**Figure 4.**
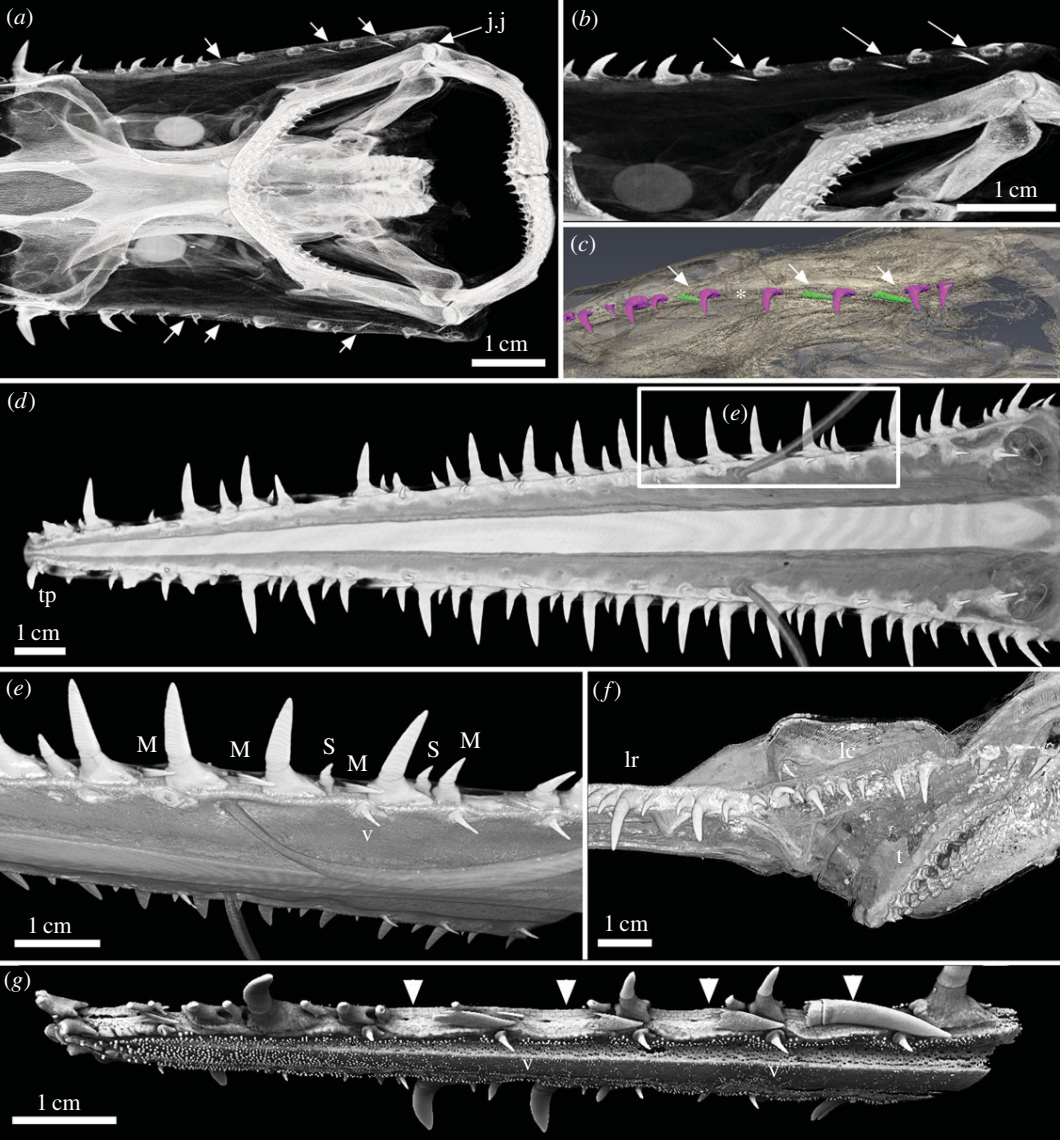
(*a*–*c*) *Pristiophorus nudipinnis* adult (Pristiophoridae; Selachii, CU unregistered specimen). (*d*–*g*) *Pristiophorus cirratus*adult (Pristiophoridae; Selachii, CU unregistered specimen). (*a*,*b*) Lateral cephalic denticles, including large and small ones (present in the embryo) ending at the jaw joint (j.j), several replacing denticles (arrows) added when a space becomes available in the series. (*c*) False-coloured render (Avizo) showing replacing denticles (white arrows) as well as large cephalic denticles (purple). Gap is present between two large denticles where the next replacement denticle would form (asterisk). (*d*) Ventral view of entire rostrum (box is region in (*e*)). (*e*) Oblique lateroventral view, erect lateral rostral denticles and addition to the series of developing crowns for ‘triplets’ and ventral rostral denticles. (*f*) Lateral view of head showing the position of the lateral cephalic denticles relative to mouth. (*g*) Lateral view of rostrum showing four replacing denticles (arrowheads) each with different stages of formation of crown and pedestal (most proximal is oldest). Note pore openings at the base of the cartilaginous embayments associated with replacing denticles. The figure is arranged with proximal to the right, dorsal to the top. tp, terminal pair of denticles; S, smaller denticles; M, medium-sized denticles; lc field, lateral cephalic field; lr, lateral rostral series of denticles; t, oral teeth; v, denticles of the ventral series.

**Figure 5.**
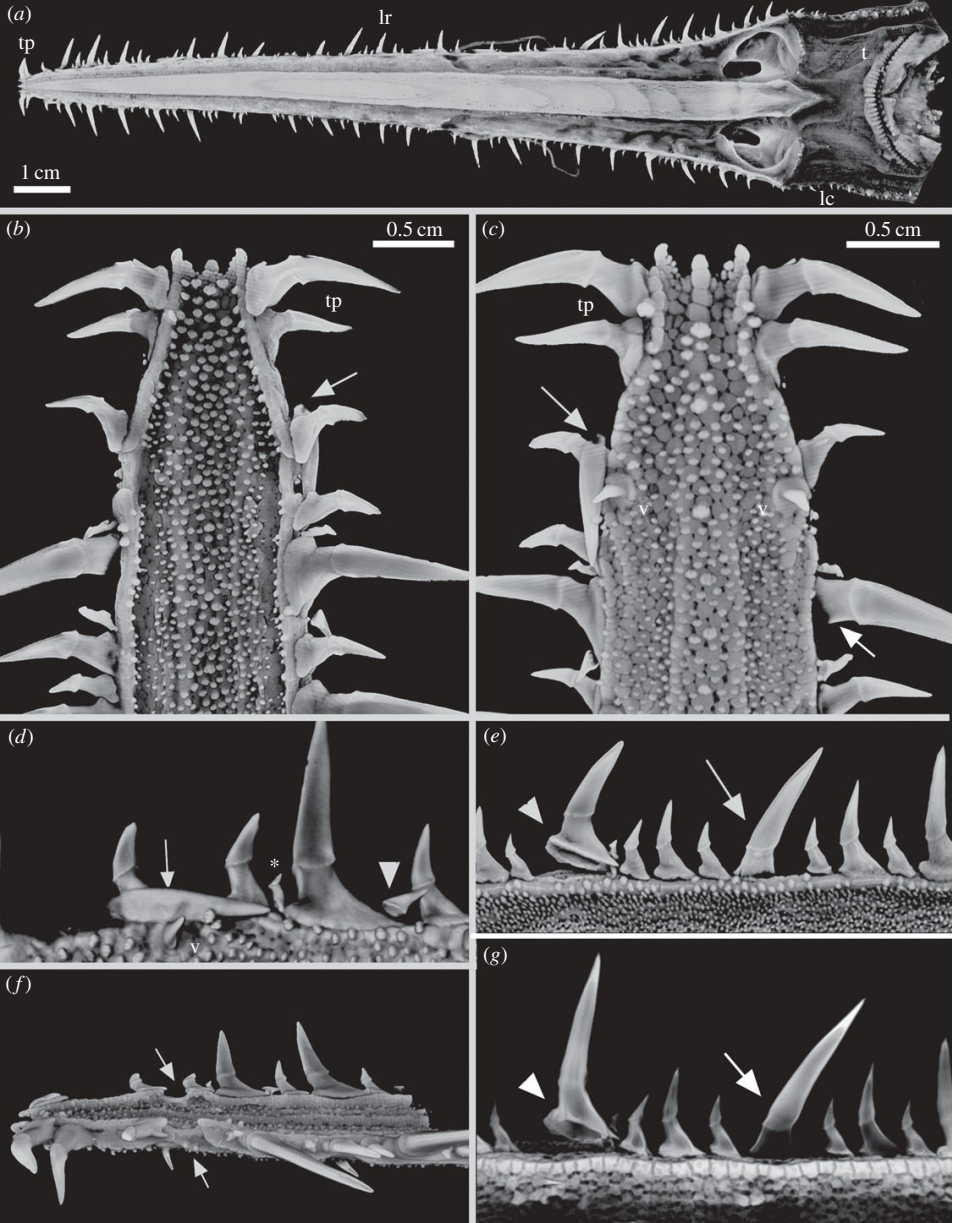
(*a*–*g*) *Pristiophorus lanae* adult (Pristiophoridae; Selachii, CU unregistered specimen). (*a*) Skull and rostrum in ventral view, comparative topography of oral teeth, with rostrum denticles of saw (lateral rostral, lateral cephalic). (*b*,*c*) High-resolution volume-rendered scans (dorsal and ventral views) of the rostrum tip, supporting two distinct, curved denticles (terminal pair); large developing denticle (arrow) and ventral denticles. (*d*) Lateral rostral denticle row, in ventral view, with covering of small, skin denticles; ventrolateral position of both large and small replacement denticles (arrow, arrowhead) confined to space between lateral and ventral ones; very small denticle in a gap between the bases of larger and medium denticles (asterisk), different from ventral denticles and small round skin denticles. (*e*,*g*) Two large denticles, different states of attachment to cartilage (arrowhead, arrow). Denticle indicated by arrowhead in process of being lost as fibrous tissues attaching the denticle within the cartilaginous pit are lost. Arrow indicates replacement denticle, partly erect with forming pedestal and base (arrow, figure 10*b*). (*g*) Same as (*e*) but with relative densities selected in Drishti to remove the skin denticle layer and reveal large prisms of cartilage support layer. (*f*) Lateral view of rostrum tip, showing a deep groove passing from the underside of the rostrum to its lateral edge (arrows). The figure is arranged with proximal to the right, dorsal to the top. tp, terminal pair of denticles; lr, lateral rostral series of denticles; t, oral teeth; lc, lateral cephalic series of denticles; v, denticles of the ventral series.

To address the question of whether the first set of lateral rostral denticle crowns in the embryo (figures 1 and 2) corresponds to those of the largest, medium or smallest denticles in the lateral rostral series on the juvenile and adult sawshark rostrum (figures 3–5), measurements of the developing crown and that of the completed tooth (all small, medium and large denticles) were taken using the three-dimensional length measurement feature of Avizo software. The results showed that the first set of lateral denticles in the embryo correspond to the large set of lateral rostral denticles in the adult (electronic supplementary material, figures S1–S3).

### 5.3 Denticle series in the adult rostrum

#### 5.3.1 Lateral rostral series

In all of the observed *Pristiophorus* specimens (*Pr. cirratus*, *Pr. nudipinnis*, *Pr. lanae*), no new series of denticles have been added relative to those in the embryo (figures 3–5).

The most significant difference between the denticle arrangement of adult specimens and the embryonic condition is that the lateral series of denticles includes denticles of different sizes. These are seen to be developing in the new spaces between the largest denticles as the rostrum grows (figures 3, 4*d*,*e*,*g* and 5). These lateral denticles are organized in discrete groups along the rostrum (‘triplets’: small, medium, large), with the largest denticles being separated by medium and smaller ones (e.g. figures 3*a*,*b*,*d*,*g*, 4*d*,*e*,*g* and 5), although this pattern appears more irregular in some specimens (figures 4*d* and 5*a*). These lateral denticles have a horseshoe-shaped base (base), supported on the cartilaginous rostrum by distinct attachment tissue (figure 10*a*, at. tiss). The base is separated from the elongate, pointed crown (cr) by dentine tissue (pedestal, ped), with other smaller denticles bases interlocking into these (figures 3*b*,*d*, 4*e*,*g* and 5*d*,*e*).

#### 5.3.2 Denticles of the rostrum tip

As noted in the embryo (developing overlapped denticles separated from more caudal denticles by a discrete gap), the rostral tip is anatomically distinct from the rest of the rostrum; two larger curved, tusk-like denticles are present, differing from the more caudal denticles, which are either curved but smaller, or larger and straighter. These are identified as the terminal pair of denticles, related to the crowded denticles at the rostrum tip in the embryo (tp, figures 4*d* and 5*a*–*c*). The cartilage supporting the most rostral denticles narrows relative to the more caudal rostrum and lacks the individual shallow depressions seen more caudally (figure 5*b*,*c*). The more caudal gap present in the *Pristiophorus* embryos, in which new denticles were developing and mineralizing (figures 1 and 2), appears to be absent, such that the only new denticles added are replacing denticles.

Near the rostrum tip are two distinct grooves (figure 5*f*, white arrows), running from the ventral to the dorsal surface. These must have held sensory structures (nerves, blood vessels), associated with the rostral tip.

#### 5.3.3 Denticles of the ventral rostral series

Denticles in functional positions have thin, narrow crowns and a round, pedestal-like base (figures 3*a*,*b*,*d*,*e*, 4*e*,*g* and 5*c*, v), are directed slightly laterally and located in small, round depressions in the mineralized rostral cartilage (figure 3*g*, white arrowheads). New ventral denticles only develop in empty cartilage depressions and are distinct, with crowns oriented caudally, and lacking a mineralized base (as in the developing lateral series; figure 3*a*,*b*, white arrows). As there appear to be a full complement of ventral denticles in the embryo, the generation of space for development of new denticles in the adult must be the result of loss. Replacement denticles are a similar size to preceding ones, with the ventral rostral denticles being a similar size in the embryo and adult and hence far smaller in the adult relative to the size of the rostrum.

#### 5.3.4 Denticles of the lateral cephalic series

**Figure 6.**
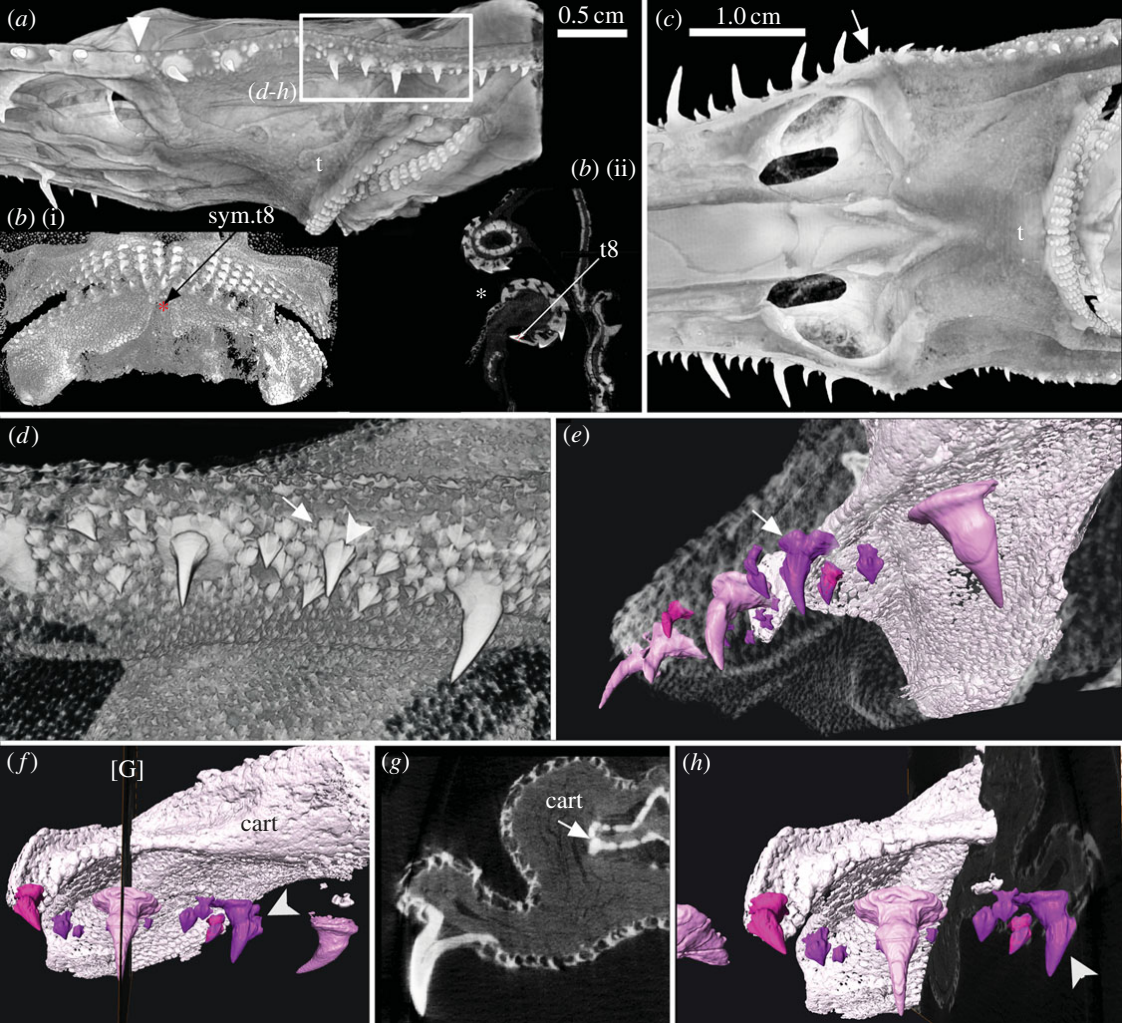
*Pristiophorus lanae* adult (Pristiophoridae; Selachii, CU unregistered specimen). (*a*) Skull and proximal rostrum in lateral view. Arrowhead indicates proximal end of rostral cartilage. Box indicates lateral cephalic denticles shown in images (*d*–*h*). B_1_, oral dentition of juvenile *Pr. lanae*, with position of section plane (B_2_). Virtual section through both jaws at symphyseal tooth file in lower jaw (sym. t8), upper jaw two parasymphyseal files, newest mineralized tooth t8 (B_2_, asterisk, mouth opening). (*c*) Skull and proximal rostrum in ventral view (arrow, junction of lateral cephalic with lateral rostral denticles). (*d*) High-resolution scan of lateral cephalic series overlain with skin denticles (arrow), larger denticles grade in size and morphology with cephalic series (arrowhead). (*e*,*f*,*h*) Avizo segmented, same denticle field (*d*), including lateral cephalic series (pink) and skin denticles (purple) relative to the cartilage of the skull (palatoquadrates, cart). One skin denticle approaches the lateral cephalic denticles in size and morphology (arrow, *e*, arrowhead, *f*,*h*), but retains typical fluting of the crown margin. (*g*) Virtual section through lateral cephalic denticle ([G] in *f*), smaller skin denticles in the skin overlap base of the lateral cephalic denticle. The figure is arranged with proximal to the right, dorsal to the top. t, oral teeth; cart, cartilage.

These denticles extend from the proximal end of lateral rostral series near the base of the rostrum, to the corner of the mouth, forming a series that gradually points ventrally rather than laterally (figures 4*a*–*c*,*f*, 5*a* and 6*a*,*c*–*h*). The shape of the base is oval to horseshoe-shaped (e.g. figure 4*a*–*c*), and these denticles are more strongly curved than those on the ventral and more distal rostrum (figures 4*a*–*c*,*f* and 6*a*,*c*). The lateral cephalic denticles are surrounded by head denticles of various sizes (figure 6*a*,*d*), these are always smaller and scattered within the skin and have morphologically different crowns with a ridged, fluted crown not seen in the smoother crowns of the lateral cephalic series (figure 6*e*, white arrow, *f*,*h*). None of the denticles in the lateral cephalic series are supported by cartilage of the head (figures 4*a*,*b* and 6*f*,*g*, cart), but would have had a fibrous attachment to the dermis, above which small skin denticles surround and overlap the bases of lateral cephalic (figure 6*a*,*d*). In a sub-adult specimen of *Pr. lanae* (figure 6), some of the skin denticles are notably larger, approaching the size of the lateral cephalic denticles while retaining a fluted crown base (figure 6*d*,*e*, white arrows). These fluted denticles are characteristic of the skin in the head region of the sawshark rostrum (figure 6*d*–*h*).

### 5.4 Denticle addition and replacement on the adult rostrum

A distinguishing feature of the sawshark rostrum is that the denticles of the lateral, ventral and cephalic series are replaced in an ordered way, but only when functional denticles are lost and space becomes available (e.g. figure 5*e*,*g*, arrowhead, a large denticle in the process of losing its fibrous attachment to the mineralized cartilaginous support tissue of the rostrum). The size of this space relates to the size of the denticle, for example, large denticles are only replaced by large denticles in the lateral rostral series, comparably sized denticles replace those in the ventral series (figures 3*b*,*d*,*f*,*g*, 4*e*,*g* and 5*b*–*e*,*g*, arrows). In the lateral rostral series, replacement denticles developing under the skin can be identified by means of their mineralized enameloid cap, absence of a pedestal and base, and their horizontal position relative to the established or functional row (figures 3*d*,*f*, 4*e*,*g* and 5*c*–*e*,*g*). Each is at a different stage of development; the enameloid-covered crown forms initially (mineralizing from tip), followed by the dentine pedestal (e.g. figures 4*g*, arrowheads and 10*a*,*b*). The replacement denticles change their orientation gradually, shifting laterally to occupy the empty depression, after which the pedestal is completed and a base forms (e.g. figures 4*g*, arrowheads and 5*c*–*e*,*g*, arrows).

Small and medium denticles are positioned on either side of these large denticles and are initially absent in the early embryo. Medium-sized denticles can be seen developing in figure 4*e* (M), just caudal to a larger denticle, although a smaller denticle can be interspersed between these (S), often within the horseshoe-shaped base of the larger denticle (also figures 3*a*,*b* and 5*d*, asterisk, and newly developing small denticle indicated by white arrowhead). New small and medium denticles also develop flat within the skin, elevating into lateral positions along the rostrum. As with the larger denticles, the bases of the smaller and medium-sized denticles develop after elevation (figures 4*e* and 5*d*). A new denticle only forms when a space becomes available due to the loss of the functional denticles (e.g. figures 4*e* and 5*d*) or due to growth of the rostrum. Replacement of the largest denticles is size specific and like-for-like. The first formed lateral rostral denticles in the embryo appear to be spatially related to the largest denticles in the adult (electronic supplementary material, figure S3). Loss of these denticles through ontogeny allows progressively larger denticles to develop, each related to a shallow depression on the lateral edge of the rostral cartilage. Thus, where the shallow depression in the cartilage is present in the lateral series, it is filled by development of a large denticle, rather than several of the smaller or medium-sized denticles (figures 4*g* and 5*b*–*d*; we predict that in the large space opposite the replacement denticle in figure 5*b*,*c* (arrow), another large denticle would have formed). Other denticles form wherever space is available and their size is related to available space rather than ontogenetic stage. Additionally, the new large denticle develops in association with foraminae in the cartilage, presumably for blood supply to the denticle pulp cavity (figure 3*f*, canals passing through rostral cartilage, figure 4*g*).

By comparison, in the ventral series, denticles are also replaced, and again, not until a space is available in the circular pit (figure 3*b*,*g*). New denticles, lying flat against the rostral cartilage, are of the same size as the previous functional denticles, and the base develops subsequently after they move into the vertical functional position (figure 3*a*,*b*). In the more caudal lateral cephalic series, denticles are also replaced, in a manner comparable to those in the lateral rostral series. That is, new denticles are only added when a space is available and initially are lying flat against the rostrum (figure 4*a*–*c*, white arrows).

## 6. Pristidae (sawfish; Batoidea)

### 6.1 Denticle series in the adult rostrum

**Figure 7.**
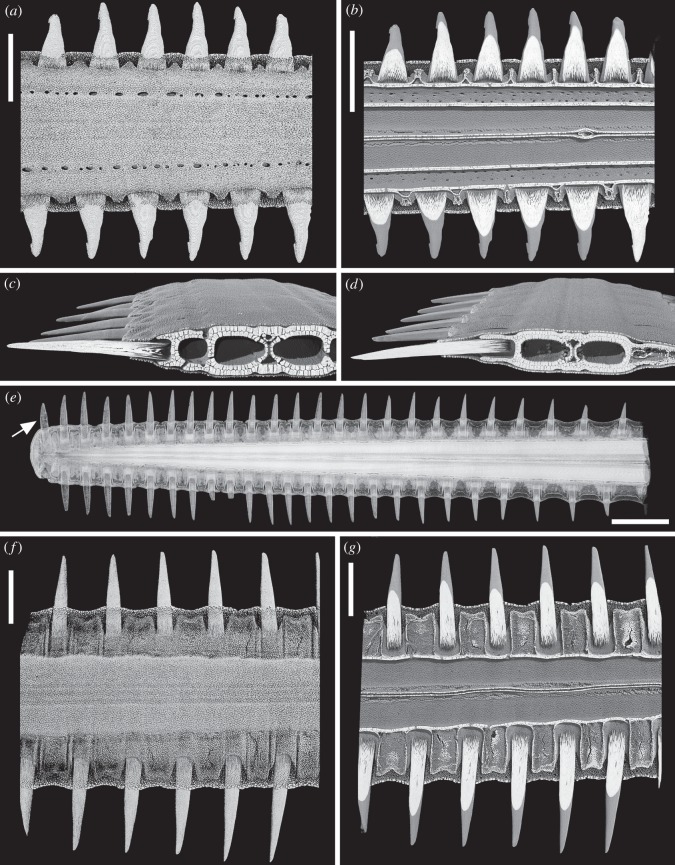
Pristidae (Batoidea). (*a*–*c*) *Anoxypristis cuspidata*, neonate rostrum. (*a*) Denticles from mid-rostrum, dorsal view. (*b*) Virtual horizontal section of mid-rostrum, dorsal view. (*c*) Virtual coronal section, mid-rostrum, shallow socket formed of prismatic cartilage. (*d*–*g*) *Pristis* sp., rostrum of neonate. (*d*) Virtual coronal section, mid-rostrum, deeper socket than (*c*). (*e*) Rostrum in dorsal view, note no paired terminal denticles (arrow, see figure 5*a*–*c*). (*f*) Mid-rostrum, dorsal view, slender tall denticles compared with those in (*a*), all are very evenly spaced, in deep ((*a*), shallow) embayments in cartilaginous rostrum. (*g*) Virtual horizontal (proximo-distal) section, mid-rostrum, dorsal view. Scale bars, 0.5 cm. The figure is arranged with proximal to the right, dorsal to the top.

In *Pristis* and *Anoxypristis*, the only denticle series present is along the lateral rostrum; lateral cephalic and ventral rostrum denticles are absent (figure 7). Miller 15 noted that in embryos of *Pristis*, the adult number of denticles had already been set, with a similar size gradation, with the largest in the middle third of the rostrum. This suggests that new denticles could be added caudally, near the base of the rostrum or closer to the tip, but only more embryonic material will confirm this. The lateral rostrum denticles are all approximately the same size other than some smaller ones near the proximal end. They are very large and are located in sockets of mineralized cartilage along the side of the rostrum. In *Anoxypristis*, rostrum denticles are stouter, have a small barb at their tips (in juveniles but lost later in ontogeny) and are held in shallower sockets compared to *Pristis* (figure 7*a*–*c* versus  7*d*–*g*). In both taxa, there is no indication that the lateral rostrum denticles are replaced in the manner described for the sawsharks, but instead the denticles grow continually from the open base as new dentine is deposited (figure 7*b*–*d*,*g*). An enameloid layer was recognized, but this is exceptionally thin and rapidly removed owing to wear on the erupted portion of the denticle. Denticles of adults often show intense wear, with continuous growth maintaining the size of the denticle relative to the (growing) rostrum.

## 7. Sclerorhynchoidea (Batoidea)

**Figure 8.**
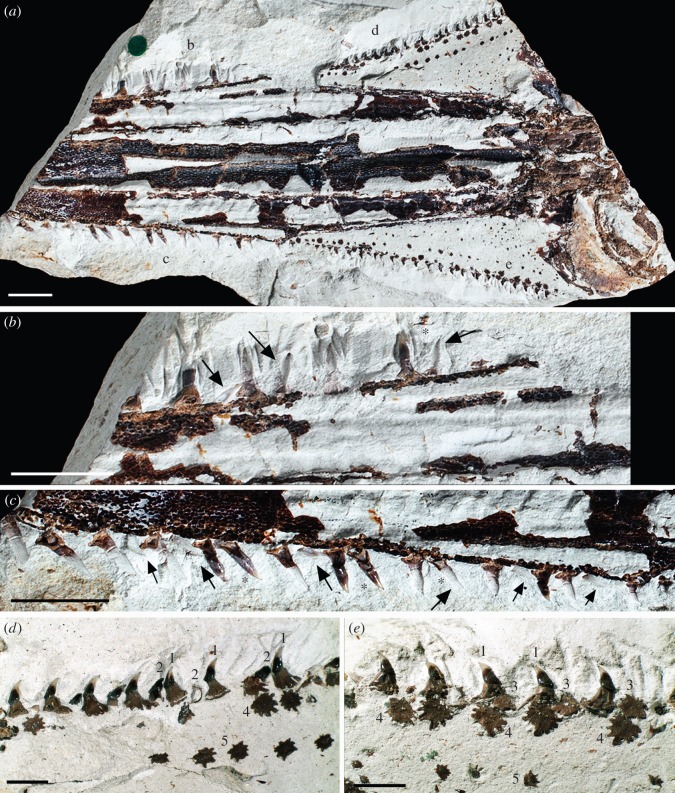
*Sclerorhynchus atavus* (Batoidea), NHMUK PV P4776. (*a*) Dorsal view of partial rostrum, skull and nasal capsules with lateral rostral (*b*,*c*) and lateral cephalic (*d*,*e*) denticle series. (*b*,*c*) Close-up of lateral rostral series, black arrows indicate replacing denticles. The asterisk indicates existing and replacing denticles forming pairs. (*d*,*e*) Close-up of lateral cephalic series, showing associated sets of denticles including the main functional row (denticle ‘1’), and series of recumbent replacing denticles (‘2’) and three series of bases visible in dorsal view (denticles ‘3–5’), representing additional ventral denticles associated with the lateral cephalic series, absent in pristiophorids. The figure is arranged with proximal to the right, dorsal to the top.

**Figure 9.**
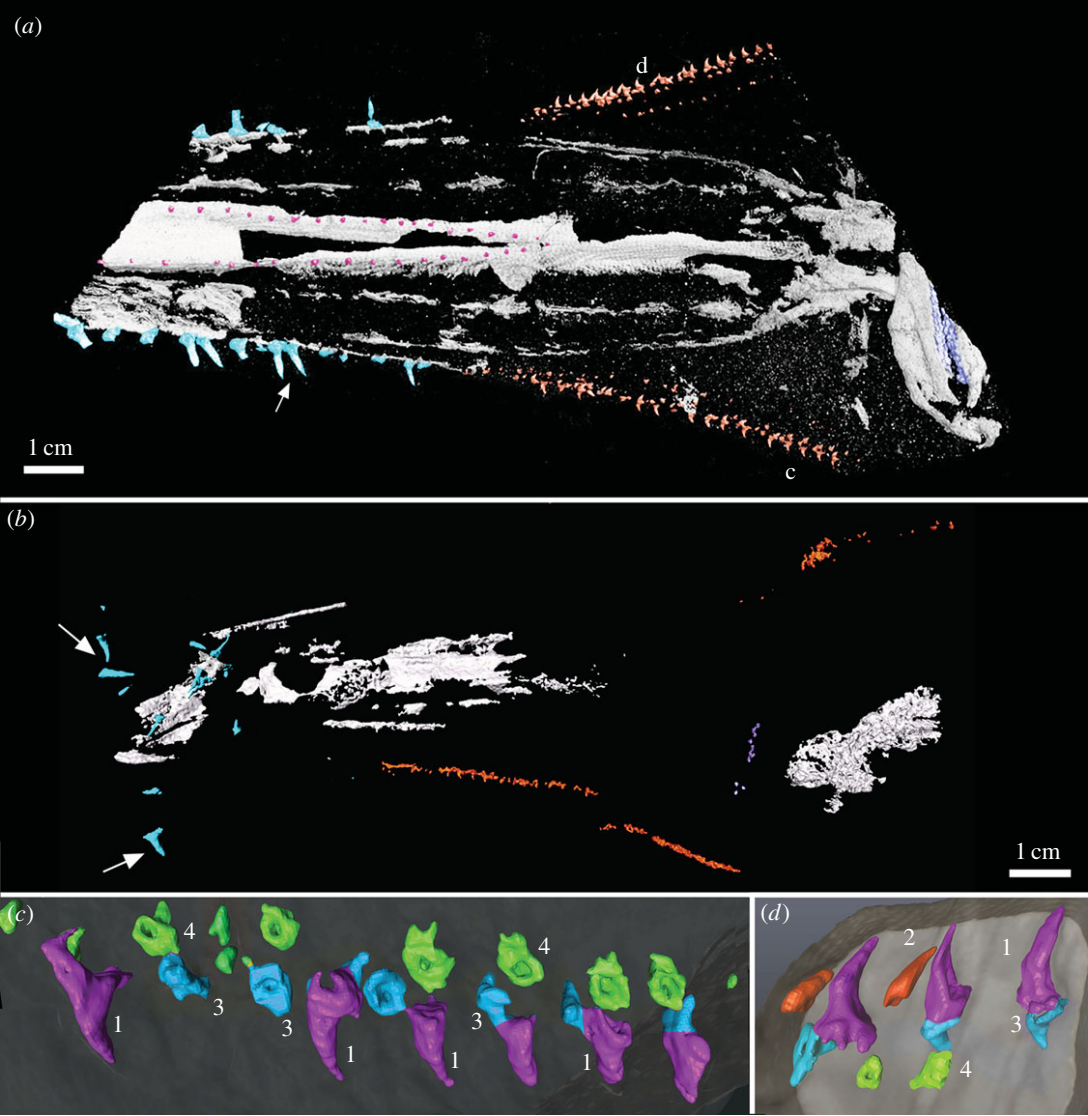
*Sclerorhynchus atavus* (Batoidea), (*a*,*c*,*d*) NHMUK PV P4776, (*b*) NHMUK PV OR83663. (*a*,*b*) Rendered images as segmented denticle series, showing lateral rostral (blue), ventral (pink) and lateral cephalic series (orange). Arrows in (*b*) indicate lateral rostral denticles disarticulated from the rostrum post-mortem. (*c*,*d*) Sets of denticles including functional (1, purple), replacing recumbent denticle (2, red) and a third closely associated to the functional denticle (3, blue). Denticles 3 and 4 (green) represent the ventral denticles associated with the lateral cephalic series. The figure is arranged with proximal to the right, dorsal to the top.

*Sclerorynchus atavus* (Cretaceous, Lebanon; figures 8 and 9) is a fossil chondrichthyan with several ray-like characters, particularly in the shape of the body, pectoral fins and position of the gill slits, assigning this species to the Batoidea 16; 17; 18; 19. Despite this phylogenetic position, the rostrum and the rostral dentition are very different from the batoids *Pristis* and *Anoxypristis* 16; 17. There are three distinct series of denticles in *Sclerorhynchus*, one associated with the rostrum, and one extending caudally along the side of the head, comparable to the lateral rostral and lateral cephalic denticles described above for the pristiophorids (figures 8 and 9). A ventral rostral series is also present in *Sclerorhynchus*, previously unrecognized (figure 9*a*, pink dots in midline, converging caudally).

In *Sclerorhynchus*, denticles in the lateral rostral series are supported by shallow depressions in the cartilage, becoming easily separated from the rostrum post-mortem (compare denticles (light blue) in figure 9*a*,*b*). The lateral rostral denticles are of approximately uniform size, with denticles of other sizes separating these being absent (‘triplets’ are absent; figure 8*a*–*c*). However, as in *Pristiophorus*, these lateral denticles are replaced and/or added to; the replacement denticles are recumbent against the rostrum, are similar in size to existing denticles, and move into a lateral, functional position (figure 8*b*,*c*, black arrows). Replacement denticles are added to open spaces along the rostrum, but these are more closely associated to existing functional denticles than in *Pristiophorus*, such that the existing and replacing denticles appear to form pairs (figure 8*b*,*c*, asterisks, 9*a*, white arrow).

The lateral cephalic series continues caudally from the lateral rostral series, but marks a distinctive change in denticle morphology (figures 8*a*,*d*,*e* and 9). The lateral cephalic series is separate from the rostral cartilage at this point, as in the pristiophorids. The functional denticles in this series are smaller than those of the lateral rostral series, are more strongly curved, with a small crown, short pedestal and a distinctive large, flaring, sinusoidal base (figures 8*d*,*e* and 9*c*,*d*, denticle ‘1’). Between these are replacing denticles, recumbent and oriented posterolaterally (figures 8*d*,*e* and 9*d*, denticle ‘2’). Two rows of denticles are positioned in very close association to the functional denticle, with comparable crowns and bases (figures 8*d*,*e* and 9*c*,*d*, denticles ‘3, 4’), but oriented ventrally relative to the functional and replacing denticles. A third series of comparably oriented denticles is located more medially (figure 8*d*,*e*, denticle ‘5’).

## 8. Oral dentitions

**Figure 10.**
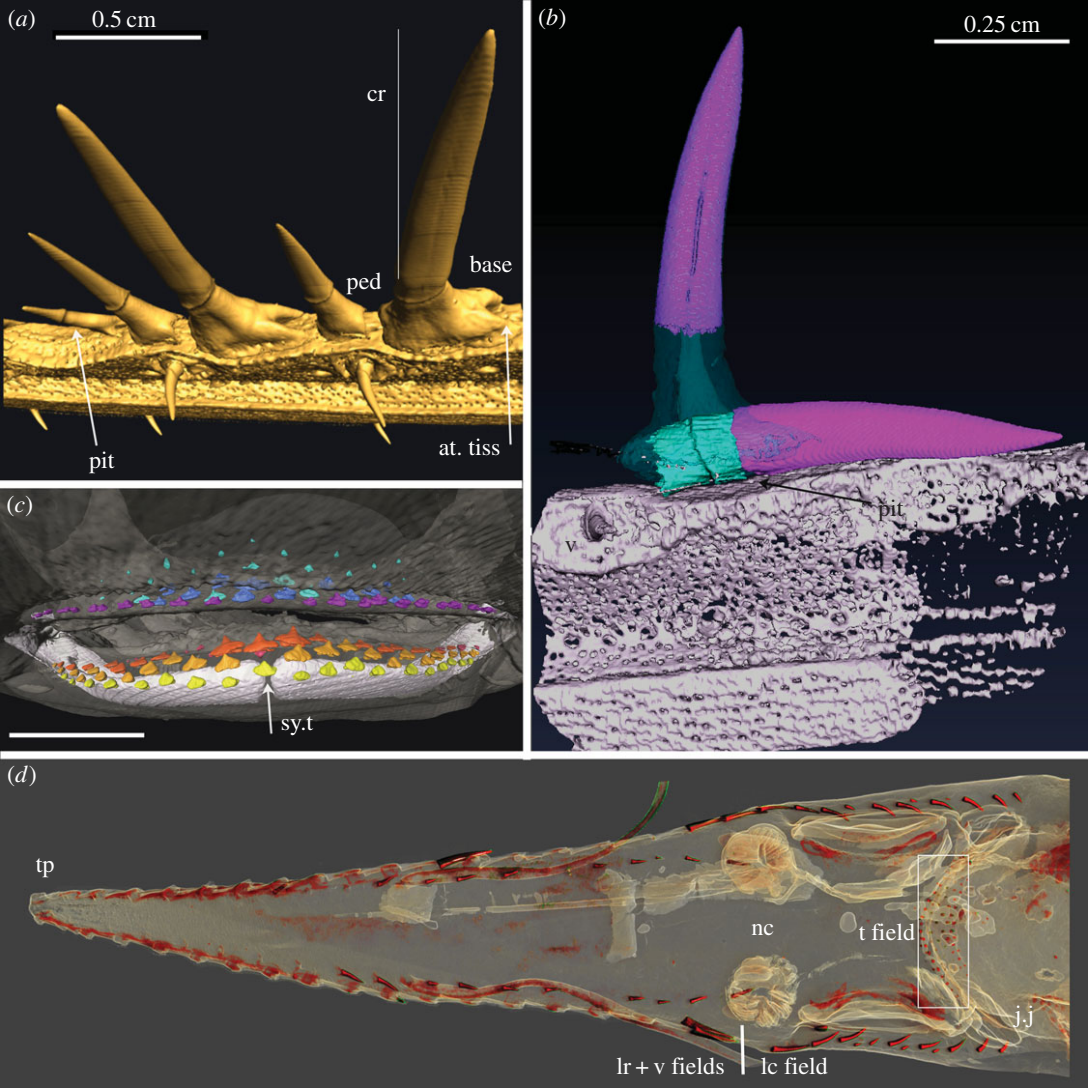
Pristiophoridae; Selachii. (a,*b*) *Pristiophorus nudipinnis* (CU unregistered specimen), adult lateral rostral and ventral denticles at 10 μm resolution, volume rendered ((*a*) Drishti) and segmented ((*b*) Avizo). (*a*) Topology of tissue arrangement for denticle attachment in shallow concave pit, in surface of support layer of prismatic cartilage (after denticle loss), empty pit to left. (*b*) Denticle elevation from horizontal to functional erect state, superimposed segmented images, enameloid crown tissue (pink), dentine (turquoise), prismatic cartilage below. (*c*) *Pristiophorus nudipinnis* BMNH1909.3.28.13, embryonic oral teeth in labial view, lower jaw with three successional rows segmented (yellow, orange and red) forming aligned files (tooth replacement series), earliest teeth with symphyseal tooth (arrow, yellow). (*d*) *Pristiophorus cirratus* (BMNH Haslan collection, unregistered), embryo, ventral view from head to rostrum tip, volume rendered (Drishti, red for highest density, mineralized tissue), illustrates four different morphogenetic fields; lateral rostral and ventral denticles, starting from nasal capsule to tip, lateral cephalic denticles from nasal capsule to level of jaw joint, oral teeth restricted to mouth (starts from midline symphysis, symmetrical addition towards right, left jaw joints, as in (*c*)). The figure is arranged with proximal to the right, dorsal to the top. cr, crown; ped, pedestal; base, basal tissue of denticle for attachment; pit, shallow embayment in surface of mineralized cartilage; at. tiss, tissue attaching base of denticle to cartilaginous rostrum; sy.t, symphyseal tooth; tp, terminal pair of denti- cles; lr+v fields, lateral rostral and ventral fields; lc field, lateral cephalic field; nc, nasal capsule; t field, oral tooth field; j.j, jaw joint.

The development of oral dentitions was observed in embryos and adults of the Pristiophoridae (figures 1*a*,*b*, 6*a*–*c* and 10*c*–*d*). In the embryo of *Pliotrema*, four to five rows of offset tooth files are present in both jaws, with tooth development (shown as crown mineralization) proceeding proximally along the jaw (figures 1*a*,*b* and 10*c*,*d*). There is a symphyseal tooth in the first tooth row in the lower jaw, but not the upper (figures 6*b*_1_ and 10*c*), where it fits between two parasymphyseal teeth. In this specimen, at the juvenile stage up to eight teeth have formed in one tooth file, arranged in single file, the latest one to form in the symphyseal file deep on the lingual side (in the dental lamina), has a mineralized enameloid cap and open pulp chamber (figure 6*b*_1_, arrow, sym.t8). The oldest tooth abuts onto the dermal denticles at the mouth margin (figure 6*b*_1,2_, asterisk, small size skin denticles), in some files the oldest teeth have gone beyond the functional surface and have not yet been shed.

## 9. Discussion

Among chondrichthyans, an extended rostrum-saw evolved independently at least five times: Holocephali (*Squaloraja palaeospondyla*, *Metopacanthus* *granulatus* 20), Pristiophoridae (Selachii), Pristidae, Sclerorhynchoidea (Batoidea) and in the fossil taxon *Bandringa rayi* (Elasmobranchii *incertae sedis* 21). Rostrum-saw denticles are lacking in *Bandringa*, but present in the other groups, albeit in a rather limited way in the holocephalans 11; 12; 13; 15; 16; 17; 18; 19; 20; 21; 22; 23; 24. Previously, Owen 22 identified the rostrum-saw denticles of *Pristis* as modified ‘dermal spines’, while Schaeffer 23 suggested they derived from modified placoid scales as also did Slaughter & Springer 11, Würinger *et al.* 19. Cappetta 24 suggested that in all three rostrum-bearing groups, including the Pristiophoridae and Sclerorhynchoidea, the sets of rostrum denticles were modified from dermal denticles. Identification as modified denticles was due to the location of these denticles on the elongate rostrum, and morphological differences with respect to the oral dentition (e.g. figures 2, 4*a*,*b* and 10*c*–*d*).

However, we aimed to show if these denticles, particularly in the Pristiophoridae and Sclerorhynchoidea, were ordered in a structural pattern with initiation, addition for succession, and positions for replacement, comparable to the tightly regulated oral dentition. In sawsharks and sclerorhynchids, we identified three differently organized regions, including lateral rostral, lateral cephalic, ventral rostral, all showing some organized initiation and replacement, differing from that of dermal denticles and synchronized with rostrum cartilage growth.

### 9.1 Initiating structural pattern in Pristiophoridae

In embryonic Pristiophoridae, rostrum-saw denticles of the lateral, lateral cephalic and ventral series develop below the skin as single denticles (figures 1*d*,*e*,*g*, 2 and 10*d*). These can be identified as separate but related developmental fields, as previously recognized by Reif 9; 25. In the embryo, all three fields converge on the nasal capsules (figures 1*d* and 10*d*), which may represent signalling centres for the origin and development of these fields. Associated with this, we propose (until more embryonic material is available to test this hypothesis) that a focal point of growth for the rostrum-saw denticles is at the tip, originally located between the two nasal capsules prior to rostrum growth. As this region develops, the distinct rostrum tip develops, where saw-denticles (within early denticle germs) are crowded together forming terminal pairs, separated from more caudal, developed denticles by a distinct gap (e.g. figures 1*g* and 2*c*–*e*). We suggest this region represents the boundary between the rostrum tip and the rest of the rostrum, where within the dermis and epithelium, a reservoir of cells is maintained for denticle initiation and morphogenesis. In figure 2*c* (white arrowheads), the crowns of new denticles are developing and mineralizing, two on one side, and one on the other, suggesting some left–right timing in development. At some stage during ontogeny, this gap is lost; the terminal saw-denticle pairs remain (figure 5*b*,*c*), and more caudally, additional spacing between the older ones occurs through interstitial growth with rostrum extension. These first saw-denticles in the lateral rostral series correspond positionally to at least most of the largest ones in the adult ‘triplet’ (electronic supplementary material), with interdental spaces filled with smaller saw-denticles of two distinct sizes; as described below, saw function is maintained by means of replacement of all.

With respect to the ventral series of denticles (those located in small pits on the ventral surface of the rostrum), they appear to be spatially associated with the large saw-denticles of the lateral rostral series, generally being positioned and initiated near them (figures 1*c*,*g*, 2*c*–*f* and 10*a*). In figure 1*c*, the ventral denticles indicated by the white arrows show less mineralized crowns than the more caudal, and the saw-denticle marked by an asterisk appears less mineralized than its opposite, again potentially an additional left–right offset in saw-denticle development. At the rostrum tip, a ventral denticle can be seen developing in association with a large saw-denticle, but absent from the gap between the tip and rostrum cartilage, where a ventral denticle would be expected to form, if developing independently from the larger lateral denticle (figures 1*c*, white arrowhead, and figure 2*e*, white arrows). This suggests that the tissues involved in development of the large saw-denticles are co-opted to form the ventral denticles.

### 9.2 Maintaining rostrum saw function by regeneration in Pristiophoridae

The developing replacement denticles of all three series repeat the stages involved in denticle initiation, with the crown developing and then mineralizing while still under the skin, with the subsequent formation of the pedestal once ‘erupted’ halfway, then the attachment base develops as the denticle moves into functional position (figures 3*a*,*b*, 4*a*–*c*,*g*, 5, 9 and 10*a*,*b*). Denticles of all three sizes are replaced, but only when a space has become available on the rostrum through denticle loss (figures 4*d*,*e*,*g* and 5*b*,*c*,*e*,*g*). There is no indication of replacement denticles forming ahead of loss of a functional denticle in pristiophorids (figure 3*f*), although in *Sclerorhynchus*, saw-denticles are in topographic association with functional ones, forming pairs (figure 8*b*,*c*). There is size specificity to the replacement in the pristiophorids, where large saw-denticles are replaced by large ones (e.g. figure 4*g*) rather than several small- or medium-sized saw-denticles. This relates to the size of the large, shallow depression in the cartilage holding the larger saw-denticles and also, space available for medium and smaller ones (e.g. figures 3*d*, 4*e*,*g* and 5*d*), rather than the relative size of the rostrum, contrary to Slaughter & Springer 11. Replacement of all denticles, only occurring when a space becomes available through detachment and loss (figures 3*f*, 5*d*,*e*,*g* and 10*a*), conforms to a general sequence where the larger saw-denticles are separated by small- and medium-sized ones (figures 3*a*,*b*,*d*, 4*e* and 5*d*), again contrary to Slaughter & Springer 11, who suggested that the size of replacing denticles was correlated with size of the rostrum. Denticles of the ventral series are also replaced only when a space is available in the shallow cartilaginous pit on the rostrum, but with a single denticle of comparable size (figure 3*a*,*b*), while saw-denticles in the lateral cephalic series are replaced in much the same manner as the lateral rostral series, including those positioned flat below the skin and rotating ventrally to erupt (figure 4*a*–*c*, white arrows).

### 9.3 Similarities with dermal denticles in Pristiophoridae

The rostrum-saw denticles of the pristiophorids show strong similarities to dermal denticles, with significant differences relative to the oral dentition. In the first instance, rostrum growth creates spaces for saw-denticle development, as can occur with dermal denticles in the skin 10. Second, creation of new saw-denticles for replacement only occurs when one in function is lost and an open space is created 26, again similar to dermal denticles 4. By comparison, teeth in the oral dentition are initiated in a regulated sequence along the jaw, mediated by genes such as *shh* and *edx* 14; 27; 28; replacement of teeth is also related to the creation of successor teeth prior to use, with the functional tooth still in place or retained over the margin of the jaw (figures 1*a* and 6*a*,*b*). As well, in the saw-denticles, there is a close, often coincident topographic relationship between enlarged skin denticles and saw-denticles (figures 1*f* and 6*a*,*h*), along with morphological and size similarities between the lateral cephalic and surrounding skin denticles. With respect to the latter similarity, absence of a distinct boundary between these and overlap of morphological fields could mediate transition between skin denticles and the lateral cephalic series in an evolutionary context.

### 9.4 Pattern organization centre

The rostrum-saw denticles do show several instances of pattern order during development. As noted above, we envisage the rostrum tip as a reservoir of odontogenic tissues for denticles at least in the Pristiophoridae and Sclerorhynchoidea, originally associated in early development with the paired nasal capsules (derived from nasal placodes, neural-crest derived). These odontogenic tissues were programmed to make larger, lateral denticles, topographically reorganized into paraxial rows along the rostrum and chondrocranium. The mechanism for change to the rostrum saw-denticle could be both a heterchronic shift from that of normal skin denticle development, to regulate timing of each different size and their same size-for-size replacement, and one of heterotopy. When the larger, lateral denticles were established, and into functional positions relative to pits in the cartilage, the odontogenic tissues present at these sites along the rostrum would later contribute to both the small and medium denticles of the rostral triplet, later to give rise to replacement denticles, but only when space becomes available by their loss. These mechanisms are also co-opted from lateral to ventral denticles on the rostrum. Features related to patterning mentioned above are like-for-like replacement of the rostrum saw-denticles, and this developmental association between the lateral rostral and ventral denticles.

Ideally, we would investigate soft tissue development of the saw-denticle germs at the earliest stages (using histological sectioning, and iodine staining before XCT) to determine whether they form from a dental lamina that is either short-lived, continuous along the jaw, or discontinuous, forming separately for each tooth generated 1; 29. Unfortunately, given the rarity of embryonic material, tooth germ development could not be examined at this point in time.

## 10. Conclusion

Our observations and comparisons of the rostrum saw-teeth to the oral dentitions demonstrate that along the rostrum, developmental order and arrangement of denticles are distinct with clear boundaries in the embryo delineating rostrum saw-teeth from that of the oral dentition (figure 10*d*). We suggest that the tooth-like denticles on the rostrum-saw were more similar, in their initiation pattern and replacement order, to that seen in generalized dermal denticles, and originated from the denticle odontogenic modules that became enlarged and arranged along the rostrum and chondrocranium, forming distinct sets in Pristiophoridae and Sclerorhychoidea. The boundaries between rostrum denticles and the small covering skin denticles are always distinct, and their pattern of replacement remains unique for each region, as one would expect from clearly established morphogenetic fields.

There is no evidence of similarity of initiation order, or replacement pattern between the rostrum-saw denticles and the regulated, timed addition and succession of their oral teeth. Key features of the organized and functional oral dentition include many successive teeth added in files in advance of tooth replacement (‘many-for-one’), to maintain structural order of the dentition. None of these features are observed in rostrum-saw denticle addition, where denticles show ‘one-for-one’ replacement triggered by loss of each, separate denticle; no new development is started before loss of each functional denticle of the rostrum-saw. These specialized rostrum-saw denticles do not possess any shared morphogenetic parameters of spacing and timing with those of the oral dentition. Our aim was to test the classical hypothesis that oral teeth are derived from external skin denticles, including a common developmental ‘toolkit’, and shared spatio-temporal patterning and ordered replacement. The tooth-like structures on the chondrichthyan rostrum were investigated in this regard, potentially showing more features in common with the oral dentition than other examples of organized skin denticles, such as the more typical placoid scales. Although there is evidence of patterning in saw-teeth along the rostrum, as described above, all regions show more similarity to the placoid scales, with respect to their initiation and replacement. Our current observations do not support an evolutionary relationship between skin denticle organization and that of oral teeth.

We conclude that denticles on each rostrum-saw are specialized denticles of the skin, selected for functional adaptability as a feeding, prey-obtaining structure. We suggest that the developmental mechanism for order (genetic regulation) of rostrum denticles could have been co-opted in evolution from other spatially patterned dermal denticles. Alternatively, these mechanisms could have been co-opted from a region associated with the nasal capsules and the anterior margin of the chondrocranium; this region initiating rostrum growth and through modification of dermal denticles (as embryonic germs) producing the ordered rostrum saw-teeth. Wider examination of less extended rostral processes of rays and sharks (e.g. the thornback ray), along with embryonic material of the pristids and pristiophorids, will allow these hypotheses to be tested. The chondrichthyan rostrum with its regulated denticles shows how the developmental module of the denticle retains significant plasticity of timing, morphogenesis, structural pattern and restricted topography, to provide the ordered denticles of the ‘saw-toothed’ rostrum.

## Supplementary Material

Sawshark rostrum denticle measurements text

## Supplementary Material

Figure S1

## Supplementary Material

Figure S2

## Supplementary Material

Table

## Data accessibility

Data available on the NHM Data Portal (http://dx.doi.org/10.5519/0056773).
