# Supplementary material for: Evolutionary origins and development of saw-teeth on the sawfish and sawshark rostrum (Elasmobranchii; Chondrichthyes)
Source: R Soc Open Sci. 2015 Sep 2;2(9):150189. doi: 10.1098/rsos.150189 (PMC4593678; doi:10.1098/rsos.150189)
Supplement: Sawshark rostrum denticle measurements text [file rsos150189supp1.docx]

**Supplementary Information**

**Teeth outside the mouth? Evolution and development of the sawfish and sawshark rostral dentitions (Elasmobranchii; Chondrichthyes)**

Monique Welten, Moya Meredith Smith, Charlie Underwood, Zerina Johanson

Slaughter and Springer [11] described the events leading to the development of the variable pattern of lateral rostral denticle length in sawsharks. X-ray images of a small number of newborn, sub-adult and adult sawsharks were used to study the pattern of lateral denticle formation and difference in sizes. They observed that newborn specimens showed a series of lateral rostral denticles that were subequal in length. After a short period of growth, a second series of lateral rostral denticles develops, about one-fourth of the length of the first set of denticles. Eventually, they found a third set of lateral denticles developing in sub-adult specimens; these denticles were about a half or two-thirds of the size of the first set of lateral denticles.

In our study, we used 3D CT scanning data and the measurement features of Avizo to study the development of the pattern of lateral denticles in different sizes in embryonic and adult sawsharks. We compare the average length of the first set of lateral denticles in embryos of *P. nudipinnis* and *P. cirratus,* with the average length of small, medium and large denticle sets of the adult specimens. The first set of lateral denticles in the sawshark embryos examined in our study consist of the enameloid crowns only; the pedestals and bases have not yet mineralised. Therefore, average length of the first set of lateral denticles in embryos was compared to the length of only the enameloid crowns of fully developed as well as developing replacement denticles (large, medium and small) in adult specimens. Average length of the first set of lateral denticles in the *P. nudipinnis* embryo is 4.99 mm, while the crowns of the smallest denticles in the adult specimen measured 1.9 mm, medium denticles 3.17 mm, and the largest 7.41 mm. Comparison of lateral denticles in *P. cirratus* embryo and adult show similar results (Supplementary Information Table 1 and Figure 1). Supplementary Information Figure 2 shows average length of first denticle set in the embryos, compared to crown length of developing replacement denticles and fully developed lateral denticles.

Since the average length of the first set of lateral denticles in the embryos is larger than the fully mineralised crowns of the small and medium lateral denticles in the adults of both species, our measurements confirm that the first set of lateral denticles in the embryo corresponds to the large denticle set in the adults, as described by Slaughter and Springer [11].

**Figure captions**

**Figure 1** Lateral denticle sets in embryonic and adult sawsharks, clustered by length (mm).

**Figure 2** Fully developed and developing lateral denticles in adult sawsharks and first denticle set in embryos, grouped by length (mm).

**Table 1** Average length of first set of lateral denticles (mm), compared to the mineralised crowns in sawshark embryos and adult specimens, respectively**.**

|  |  |  |  |  |  |  |  |
| --- | --- | --- | --- | --- | --- | --- | --- |
|  |  |  |  |  |  |  |  |
|  |  |  |  |  |  |  |  |
|  |  |  |  |  |  |  |  |
