# Supplementary figures and images for: Evolutionary origins and development of saw-teeth on the sawfish and sawshark rostrum (Elasmobranchii; Chondrichthyes)
Source: R Soc Open Sci. 2015 Sep 2;2(9):150189. doi: 10.1098/rsos.150189 (PMC4593678; doi:10.1098/rsos.150189)

**Average lateral denticle length (mm)**

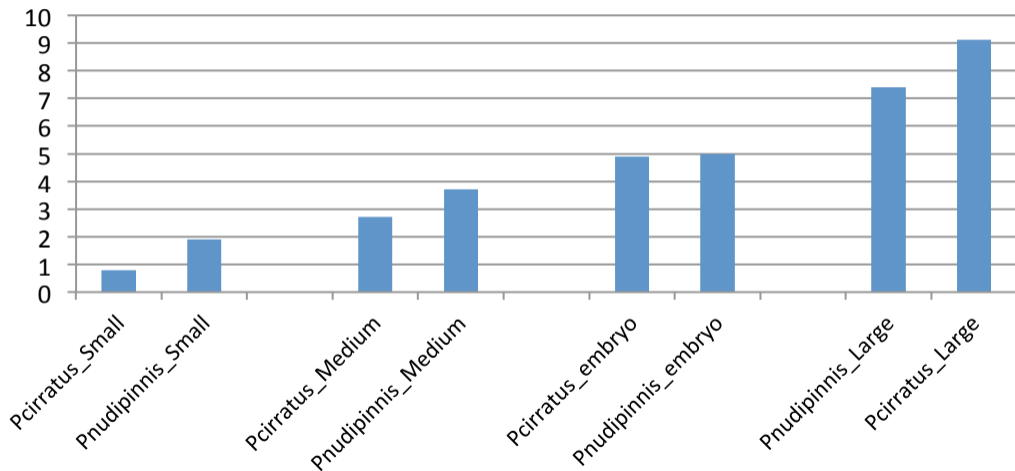

Supplement: Figure S1 [file rsos150189supp2.pdf]

**Average lateral denticle length (mm)**

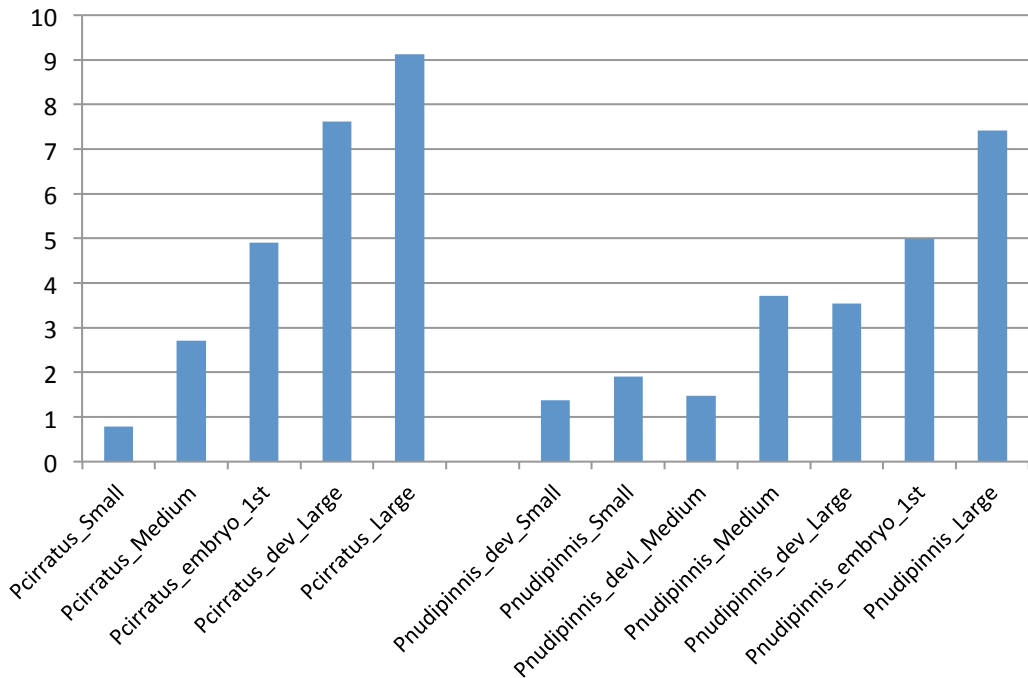

Supplement: Figure S2 [file rsos150189supp3.pdf]
