# Supplementary material for: Evolutionary origins and development of saw-teeth on the sawfish and sawshark rostrum (Elasmobranchii; Chondrichthyes)
Source: R Soc Open Sci. 2015 Sep 2;2(9):150189. doi: 10.1098/rsos.150189 (PMC4593678; doi:10.1098/rsos.150189)
Supplement: Table [file rsos150189supp4.pdf]

Specimen		Average length of mineralised crown in mm				
		1st denticle set	small denticles	medium denticles	large denticles	Rostrum length (distal tip-nasal capsules) in mm
Pristiophorus nudipinnis	embryo	4.99				76.7
Pristiophorus nudipinnis	adult		1.90	3.72	7.41	206.4
Pristiophorus cirratus	embryo	4.90				68.1
Pristiophorus cirratus	adult		0.79	2.71	9.12	275.0
